# Efficient Voronoi volume estimation for DEM simulations of granular materials under confined conditions

**DOI:** 10.1016/j.mex.2015.02.004

**Published:** 2015-02-16

**Authors:** Göran Frenning

**Affiliations:** Department of Pharmacy, Uppsala University, P.O. Box 580, SE-751 23 Uppsala, Sweden

**Keywords:** Voronoi volume, Computational efficiency, Discrete element method, Granular materials, Confined compression

## Abstract

When the discrete element method (DEM) is used to simulate confined compression of granular materials, the need arises to estimate the void space surrounding each particle with Voronoi polyhedra. This entails recurring Voronoi tessellation with small changes in the geometry, resulting in a considerable computational overhead. To overcome this limitation, we propose a method with the following features:

• A local determination of the polyhedron volume is used, which considerably simplifies implementation of the method.
• A linear approximation of the polyhedron volume is utilised, with intermittent exact volume calculations when needed.
• The method allows highly accurate volume estimates to be obtained at a considerably reduced computational cost.

## Graphical abstract

**Figure:**
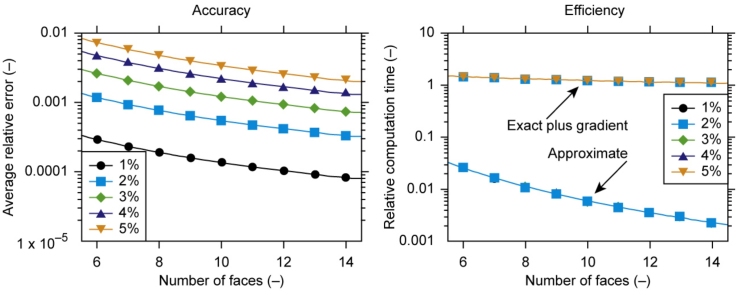

## Method details

**Fig. 1:**
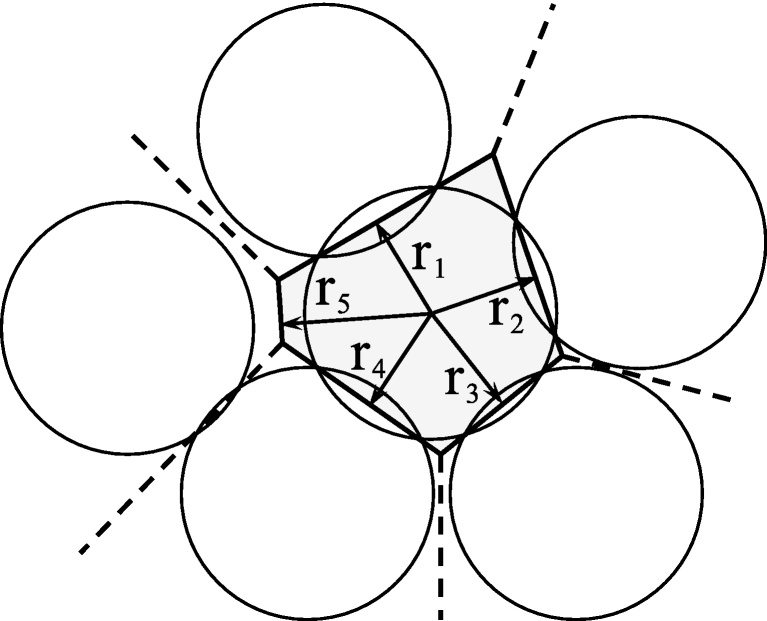
Specification of the Voronoi polyhedron (or polygon in two dimensions) in terms of the vectors **r**_*k*_, which in this case are obtained from an analysis of particle packing.

We describe a method to approximate the volume of Voronoi polyhedra in situations where recurring updates are needed for small changes in the geometry, as during simulations of confined compression of granular materials with the discrete element method (DEM) [1] (see section ‘Additional information: background’). The method utilises a linear approximation of the volume, with intermittent exact volume calculations when needed. The Voronoi polyhedron is specified in terms of *n* vectors **r**_*k*_ (with *k* = 1, …, *n*), see Fig. 1. Hence, the Voronoi polyhedron is represented by a system of inequalities,

(1) Rx≤f,

where **x** denotes the spatial coordinates, **R** is an *n* × 3 matrix whose *k*th row equals **r**_*k*_ and **f** is an *n* dimensional array with components

(2) $$f_{k}=∥{\text{r}}_{k}{∥}^{2}.$$

It is clear from (1), (2) that the polyhedron, and hence its volume *V*, is a function of **R**. Moreover, the vectors **r**_*k*_ and their magnitudes ∥**r**_*k*_∥ are routinely determined during each time step of a DEM simulation.

The polyhedron volume is known to be continuously differentiable with respect to all parameters occurring in the defining inequality system (1) [2]. Hence the volume can be approximated by the linear form

(3) $$V\approx V_{0}+{\text{G}}_{0}:\Delta \text{R}$$

where *V*_0_ is the volume at **R**_0_,

(4) $${\text{G}}_{0}={\left(\frac{\partial V}{\partial \text{R}}\right)}_{0},$$

is the gradient at **R**_0_, Δ**R** = **R** − **R**_0_ is the change in **R** relative to **R**_0_ and the colon indicates double contraction. The approximation (3) is expected to be valid provided that the change in **R** is small.

The polyhedron volume is calculated according to the recursive projection scheme proposed by Lasserre [3], which is convenient to use when the polyhedron has a halfspace representation as in (1). The details are provided in section ‘Volume calculation’ in order to define the relevant quantities for the subsequent developments. The gradient determination, described in section ‘Gradient calculation’, utilises formulae derived by Müller et al. [2] and Lasserre [4].

### Volume calculation

**Fig. 2:**
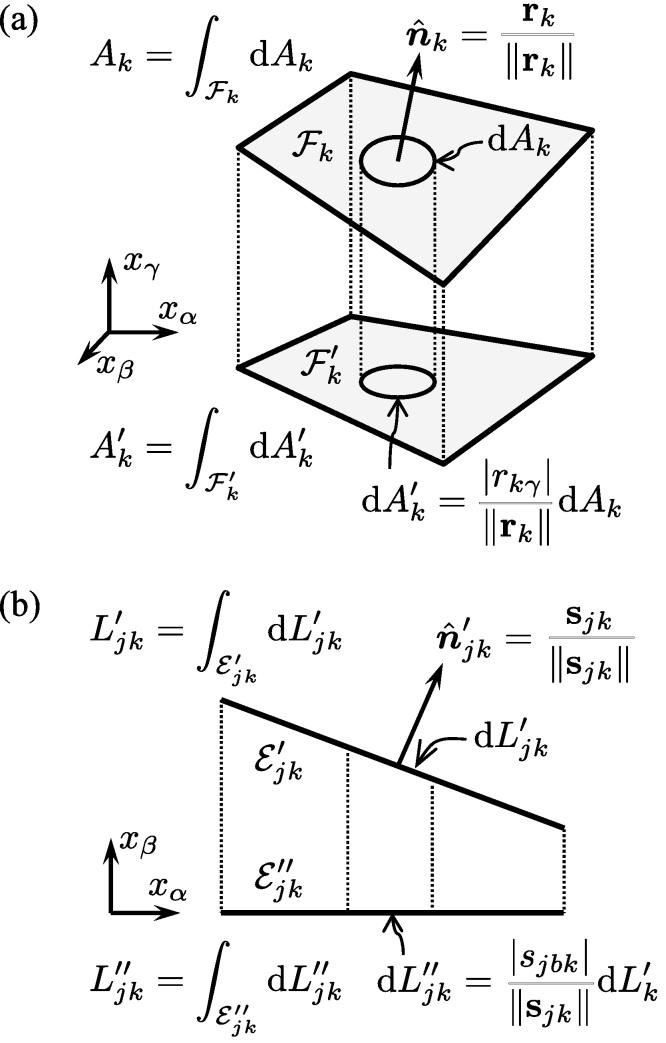
Projection of (a) face $F_{k}$ onto the plane *x*_*γ*_ = 0 (to yield $F_{k}^{\prime }$) and (b) edge $E_{\mathrm{jk}}^{\prime }$ onto the plane *x*_*β*_ = 0 (to yield $E_{\mathrm{jk}}^{\prime \prime }$).

The convex polyhedron defined by the inequality system (1) may be decomposed into *n* pyramids. Hence its volume can be expressed as

(5) $$V=\frac{1}{3}\sum_{k=1}^{n}∥{\text{r}}_{k}∥A_{k},$$

where *A*_*k*_ is the area of face *k*. Expression (5) is not useful unless a way be found to calculate the face areas. To this end, Lasserre [3] suggested a straightforward projection scheme. If we let $A_{k}^{\prime }$ denote the area of face *k* when projected onto the plane *x*_*γ*_ = 0, as illustrated in Fig. 2a, we obtain

(6) $$A_{k}^{\prime }=\frac{\left|r_{k\gamma }\right|}{∥{\text{r}}_{k}∥}A_{k}.$$

As in Fig. 2a, the unprojected and projected face *k* will henceforth be denoted by $F_{k}$ and $F_{k}^{\prime }$, respectively. For Eq. (6) to be useful, we must demand that |*r*_*kγ*_| > 0. As is commonly done [5], we select *γ* so that |*r*_*kγ*_| is maximised (although *γ* depends on *k*, we will write *γ* rather than *γ*_*k*_ for notational simplicity). Combining Eqs. (2), (5), (6), the polyhedron volume can be expressed as

(7) $$V=\frac{1}{3}\sum_{k=1}^{n}\frac{f_{k}A_{k}^{\prime }}{\left|r_{k\gamma }\right|}.$$

The next task is to determine the area $A_{k}^{\prime }$.

Eliminating *x*_*γ*_ from the inequalities (1) using the equation **r**_*k*_ · **x** = *f*_*k*_, one finds that $F_{k}^{\prime }$ is defined by a systems of inequalities of the form

(8) $${\text{S}}_{k}\text{y}\leq {\text{g}}_{k}.$$

Here, **S**_*k*_ is an *n* × 2 matrix, **g**_*k*_ is an *n* dimensional array and **y** is a vector containing the remaining two spatial coordinates. Row *j* of **S**_*k*_ may be considered as a two-dimensional vector, denoted by **s**_*jk*_, analogous to the three-dimensional vector **r**_*k*_. If we let *s*_*jmk*_ be component (*j*, *m*) of **S**_*k*_ and *g*_*jk*_ be component *j* of **g**_*k*_, we specifically obtain

(9) $$s_{\mathrm{jmk}}=r_{\mathrm{jM}}-p_{\mathrm{jk}}r_{\mathrm{kM}},$$

(10) $$g_{\mathrm{jk}}=f_{j}-p_{\mathrm{jk}}f_{k},$$

where *p*_*jk*_ = *r*_*jγ*_/*r*_*kγ*_. In Eq. (9), *M* = *m* when *m* < *γ* and *M* = *m* + 1 when *m* ≥ *γ*, to accommodate for the projection. Clearly, *s*_*jmk*_ and *g*_*jk*_ need only be determined for *j* ≠ *k*, since the inequality on row *j* in (8) will be trivially satisfied.

The special case when ∥**s**_*jk*_∥ vanishes deserves some special attention. From the two equalities embodied in Eq. (9) together with the definition of *p*_*jk*_, it is seen that ∥**s**_*jk*_∥ can only vanish if **r**_*j*_ and **r**_*k*_ are linearly dependent (parallel if *p*_*jk*_ > 0 and antiparallel if *p*_*jk*_ < 0). When **r**_*j*_ and **r**_*k*_ are parallel, the ‘outer’ constraint is redundant and shall not be included in the subsequent analysis. This constraint is in turn identified by the sign of *g*_*jk*_ (the constraint imposed by **r**_*j*_ is redundant when *g*_*jk*_ > 0 and the one imposed by **r**_*k*_ when *g*_*jk*_ < 0). Hence, **r**_*j*_ and **r**_*k*_ are parallel and **r**_*k*_ is redundant when *p*_*jk*_ > 0 and *g*_*jk*_ < 0, in which case $F_{k}$ shall not be considered further.

The convex polygon defined by the inequality system (8) may be decomposed into triangles. Hence its area may be expressed as

(11) $$A_{k}^{\prime }=\frac{1}{2}\sum_{j\in S_{k}}\rho_{\mathrm{jk}}L_{\mathrm{jk}}^{\prime },$$

where $S_{k}$ denotes the index set for the edges on $F_{k}^{\prime },L_{\mathrm{jk}}^{\prime }$ is the length of edge $j\in S_{k}$ and

(12) $$\rho_{\mathrm{jk}}=\frac{g_{\mathrm{jk}}}{∥{\text{s}}_{\mathrm{jk}}∥}$$

is the perpendicular distance from the origin to the edge. The edge length is calculated via Lasserre's projection scheme. If we let $L_{\mathrm{jk}}^{\prime \prime }$ denote the length of edge $j\in S_{k}$ when projected onto the line *x*_*β*_ = *y*_*b*_ = 0, as illustrated in Fig. 2b, we obtain

(13) $$L_{\mathrm{jk}}^{\prime \prime }=\frac{\left|s_{\mathrm{jbk}}\right|}{∥{\text{s}}_{\mathrm{jk}}∥}L_{\mathrm{jk}}^{\prime }.$$

As in Fig. 2b, the once-projected and twice-projected edge $E_{\mathrm{jk}}$ will henceforth be denoted by $E_{\mathrm{jk}}^{\prime }$ and $E_{\mathrm{jk}}^{\prime \prime }$, respectively. We select *b* so that |*s*_*jbk*_| is maximised (although *b* make take on different values for different faces $j\in S_{k}$, we will write *b* rather than *b*_*jk*_ for notational simplicity). Combining Eqs. (11), (12), (13) we find that

(14) $$A_{k}^{\prime }=\frac{1}{2}\sum_{j\in S_{k}}\frac{g_{\mathrm{jk}}L_{\mathrm{jk}}^{\prime \prime }}{\left|s_{\mathrm{jbk}}\right|}.$$

Eliminating *y*_*b*_ from (8) using the equation **s**_*jk*_ · **y** = *g*_*jk*_, one finds that $E_{\mathrm{jk}}^{\prime \prime }$ is defined by a system of inequalities of the form

(15) $${\text{t}}_{\mathrm{jk}}z\leq {\text{h}}_{\mathrm{jk}},$$

where **t**_*jk*_ and **h**_*jk*_ both are *n* dimensional arrays and *z* is a scalar. If we let *t*_*ijk*_ and *h*_*ijk*_ be component *i* of **t**_*jk*_ and **h**_*jk*_, respectively, we specifically obtain

(16) $$t_{\mathrm{ijk}}=s_{\mathrm{iak}}-q_{\mathrm{ijk}}s_{\mathrm{jak}},$$

(17) $$h_{\mathrm{ijk}}=g_{\mathrm{ik}}-q_{\mathrm{ijk}}g_{\mathrm{jk}},$$

where *q*_*ijk*_ = *s*_*ibk*_/*s*_*jbk*_ and *a* = 3 − *b* indicates the remaining component of **y**. Clearly, *t*_*ijk*_ and *h*_*ijk*_ need only be determined when *i*, *j* and *k* are all unequal.

Special considerations are needed when *t*_*ijk*_ vanishes. From Eq. (16) and the definition of *q*_*ijk*_, it is seen that *t*_*ijk*_ can only vanish if **s**_*ik*_ and **s**_*jk*_ are linearly dependent (parallel if *q*_*ijk*_ > 0 and antiparallel if *q*_*ijk*_ < 0). When **s**_*ik*_ and **s**_*jk*_ are parallel, the ‘outer’ constraint is redundant and shall not be included in the subsequent analysis. This constraint is in turn identified by the sign of *h*_*ijk*_ (the constraint imposed by **s**_*ik*_ is redundant when *h*_*ijk*_ > 0 and the one imposed by **s**_*jk*_ when *h*_*ijk*_ < 0). Hence, **s**_*ik*_ and **s**_*jk*_ are parallel and **s**_*jk*_ is redundant when *q*_*ijk*_ > 0 and *h*_*ijk*_ < 0, in which case $E_{\mathrm{jk}}^{\prime \prime }$ shall not be considered further.

It is clear from the inequality system (15) that the *least* upper bound on *z* equals the *smallest* value of *h*_*ijk*_/*t*_*ijk*_ for which *t*_*ijk*_ > 0, denoted by *u*_*μjk*_. Analogously, the *greatest* lower bound equals the *largest* value of *h*_*ijk*_/*t*_*ijk*_ for which *t*_*ijk*_ < 0, denoted by *u*_*νjk*_. When $u_{\mu \mathrm{jk}}>u_{\nu \mathrm{jk}},j\in S_{k}$ and the edge length becomes $L_{\mathrm{jk}}^{\prime \prime }=u_{\mu \mathrm{jk}}-u_{\nu \mathrm{jk}}$. Otherwise, $j\notin S_{k}$ and this combination of *j* and *k* needs not be considered further. Moreover, if there is no *t*_*ijk*_ > 0 or no *t*_*ijk*_ < 0, the polyhedron is unbounded.

### Gradient calculation

Using the chain rule, one finds that

(18) $$G_{\mathrm{kl}}=\frac{\partial V}{\partial r_{\mathrm{kl}}}={\left(\frac{\partial V}{\partial r_{\mathrm{kl}}}\right)}_{\text{f}}+2r_{\mathrm{kl}}{\left(\frac{\partial V}{\partial f_{k}}\right)}_{\text{R}},$$

where the first derivative is to be taken with the right-hand-side vector **f** in (1) fixed and the second derivative with the left-hand-side matrix **R** fixed. Using Theorem 1 in [2], the derivatives in Eq. (18) can be expressed as

(19) $${\left(\frac{\partial V}{\partial r_{\mathrm{kl}}}\right)}_{\text{f}}=-\frac{1}{∥{\text{r}}_{k}∥}\int_{F_{k}}x_{l}dA_{k}=-\frac{F_{\mathrm{kl}}}{\left|r_{k\gamma }\right|},$$

(20) $${\left(\frac{\partial V}{\partial f_{k}}\right)}_{\text{R}}=+\frac{1}{∥{\text{r}}_{k}∥}\int_{F_{k}}dA_{k}=\frac{A_{k}^{\prime }}{\left|r_{k\gamma }\right|},$$

where a variable substitution has been accomplished via Eq. (6) and where we have let

(21) $$F_{\mathrm{kl}}=\int_{F_{k}^{\prime }}x_{l}dA_{k}^{\prime }.$$

As demonstrated by Lasserre [4], integrals on faces of convex polyhedra can be expressed in terms of sums of integrals on the face edges, provided that the integrand is a homogeneous function on the face. To be able to use this result, we need to distinguish between different cases. We let {*α*, *β*, *γ*} be a permutation of {1, 2, 3}, such that the first projection is along *γ* (the value of *γ* depends on *k*) and the second projection is along *β* (the value of *β* depends on *j* and *k*).

When *l* ≠ *γ*, *x*_*l*_ indeed is a homogeneous function of degree 1 on $F_{k}^{\prime }$. Using Theorem 2.4 in [4], we thus obtain

(22) $$F_{\mathrm{kl}}=\frac{1}{3}\sum_{j\in S_{k}}\frac{g_{\mathrm{jk}}}{∥{\text{s}}_{\mathrm{jk}}∥}\int_{E_{\mathrm{jk}}^{\prime }}x_{l}dL_{\mathrm{jk}}^{\prime }=\frac{1}{3}\sum_{j\in S_{k}}\frac{g_{\mathrm{jk}}G_{\mathrm{jkl}}}{\left|s_{\mathrm{jbk}}\right|},\;l\neq \gamma ,$$

where a variable substitution has been accomplished via Eq. (13) and where we have let

(23) $$G_{\mathrm{jkl}}=\int_{E_{\mathrm{jk}}^{\prime \prime }}x_{l}dL_{\mathrm{jk}}^{\prime \prime }=\int_{E_{\mathrm{jk}}^{\prime \prime }}x_{l}dx_{\alpha },\;l\neq \gamma ,$$

noting that $dL_{\mathrm{jk}}^{\prime \prime }=dx_{\alpha }$. The case *l* ≠ *γ* can be further subdivided into *l* = *α* and *l* = *β*. When *l* = *α*,

(24) $$G_{\mathrm{jk}\alpha }=\int_{E_{\mathrm{jk}}^{\prime \prime }}x_{\alpha }dx_{\alpha }=\frac{u_{\mu \mathrm{jk}}^{2}-u_{\nu \mathrm{jk}}^{2}}{2}=L_{\mathrm{jk}}^{\prime \prime }M_{\mathrm{jk}}^{\prime \prime },$$

where $M_{\mathrm{jk}}^{\prime \prime }=(u_{\mu \mathrm{jk}}+u_{\nu \mathrm{jk}})/2$. When *l* = *β*, the second projection onto the line *x*_*l*_ = *x*_*β*_ = 0 is accomplished via the equation *s*_*jak*_*x*_*α*_ + *s*_*jbk*_*x*_*β*_ = *g*_*jk*_. Consequently

(25) $$G_{\mathrm{jk}\beta }=\int_{E_{\mathrm{jk}}^{\prime \prime }}x_{\beta }dx_{\alpha }=\frac{1}{s_{\mathrm{jbk}}}\int_{E_{\mathrm{jk}}^{\prime \prime }}(g_{\mathrm{jk}}-s_{\mathrm{jak}}x_{\alpha })dx_{\alpha }=\frac{L_{\mathrm{jk}}^{\prime \prime }(g_{\mathrm{jk}}-s_{\mathrm{jak}}M_{\mathrm{jk}}^{\prime \prime })}{s_{\mathrm{jbk}}}.$$

When *l* = *γ*, the first projection onto the plane *x*_*l*_ = *x*_*γ*_ = 0 is accomplished via the equation *r*_*kα*_*x*_*α*_ + *r*_*kβ*_*x*_*β*_ + *r*_*kγ*_*x*_*γ*_ = *f*_*k*_. Consequently

(26) $$F_{k\gamma }=\frac{1}{r_{k\gamma }}\int_{F_{k}^{\prime }}[f_{k}-(r_{k\alpha }x_{\alpha }+r_{k\beta }x_{\beta })]dA_{k}^{\prime }=\frac{1}{r_{k\gamma }}\left(f_{k}A_{k}^{\prime }-\frac{1}{3}\sum_{j\in S_{k}}\frac{g_{\mathrm{jk}}H_{\mathrm{jk}\gamma }}{\left|s_{\mathrm{jbk}}\right|}\right)$$

where Theorem 2.4 in [4] has been used (note that *r*_*kα*_*x*_*α*_ + *r*_*kβ*_*x*_*β*_ is a homogeneous function of degree 1 on $F_{k}^{\prime }$). A variable substitution has been accomplished via Eq. (13), again noting that $dL_{\mathrm{jk}}^{\prime \prime }=dx_{\alpha }$, and we have let

(27) $$H_{\mathrm{jk}\gamma }=\int_{E_{\mathrm{jk}}^{\prime \prime }}(r_{k\alpha }x_{\alpha }+r_{k\beta }x_{\beta })dx_{\alpha }=r_{k\alpha }G_{\mathrm{jk}\alpha }+r_{k\beta }G_{\mathrm{jk}\beta }=\frac{L_{\mathrm{jk}}^{\prime \prime }(r_{k\beta }g_{\mathrm{jk}}+d_{\mathrm{jk}}M_{\mathrm{jk}}^{\prime \prime })}{s_{\mathrm{jbk}}}$$

where *d*_*jk*_ = *r*_*kα*_*s*_*jbk*_ − *r*_*kβ*_*s*_*jak*_.

## Numerical simulations

Numerical simulations were performed to test the accuracy and computational efficiency of the described method. To assess the overall characteristics of the method, a set of random polyhedra was investigated. To test the performance of the method in its intended application, a small-scale DEM simulation was performed.

### Random polyhedra

Each random polyhedron was specified in terms of *n* vectors **r**_*k*_ (cf. ‘Method details’ section). The geometry was selected so that it would be relevant for volume estimation in conjunction with simulations of particulate systems with the DEM. It was assumed that all particles had the same diameter *d*_0_ (corresponding to the radius *r*_0_ = *d*_0_/2) and that the maximal particle–particle overlap was *δ*_**max**_, corresponding to a minimal dihedral angle between the vectors **r**_*k*_ of

(28) $$\varphi_{\text{min}}=\arccos\left[1-\frac{1}{2}{\left(1-\frac{\delta_{\text{max}}}{d_{0}}\right)}^{2}\right].$$

The value of *δ*_max_/*d*_0_ was kept fixed at 0.2, corresponding to a minimal dihedral angle *φ*_max_ of about 47°. First, *n* random unit vectors ${\hat{\text{n}}}_{k}$ were generated, such that the dihedral angle between any pair was at least *φ*_min_. Specifically, the components of **n**_*k*_ were drawn from independent uniform distributions on the interval [−1,1], and ${\hat{\text{n}}}_{k}$ was calculated as **n**_*k*_/∥ **n**_*k*_ ∥ whenever ∥**n**_*k*_ ∥ >0. Thereafter, **r**_*k*_ was calculated as $r_{k}{\hat{\text{n}}}_{k}$, where the magnitude *r*_*k*_ was drawn from a uniform distribution on the interval [*r*_0_ − *δ*_**max**_/2, *r*_0_]. Keeping the intended application in mind, only polyhedra with volumes not exceeding (3/2)*V*_**sph**_ were included in the subsequent analysis, where $V_{\text{sph}}=\pi d_{0}^{3}/6$ is the particle volume. Components of Δ**R** were drawn from a uniform distribution on the interval [− *ρ*, + *ρ*], with *ρ*/*r*_0_ ranging from 1 to 5%.

For each value of *n* between 6 and 14, 1000 random polyhedra were generated as described above. For each random polyhedron and value of *ρ*, 1000 different variations Δ**R** were generated, and for each variation, both the exact (*V*) and approximate (*V*_approx_) Voronoi volumes were determined. As error estimate, the relative error of the Voronoi volume was calculated as

(29) $${ɛ}_{V}=\frac{V_{\mathrm{approx}}-V}{V}$$

and the average and maximal values of |*ɛ*_*V*_| were determined. The computation times needed for full (i.e., exact plus gradient), exact and approximate volume determinations were recorded. A special routine implemented according to the description in section ‘Volume calculation’ were used for the exact volume determinations.

### DEM simulation

Uniaxial compression of 100 spherical particles (diameter 1.0 mm) in a cylindrical die (diameter 5.0 mm) was simulated with the DEM [1]. The initial particle bed had a height of about 5.25 mm and the upper punch was lowered at a rate of 5 mm/s for a period of 0.52 s until a nonporous compact was formed. For illustrative purposes, a standard contact model of the linear spring–dashpot type was used as described in [6]. The particle density was 1.45 g/cm^3^ and the normal and tangential stiffness were 100 N/mm. The sliding and rolling friction coefficients were 0.5 and 0.001 for contact between particles whereas five times smaller values were used for contact between particles and confining surfaces. The normal and tangential damping coefficients were chosen so that the fractional damping was 0.3. The time step was 0.138 μs, implying that about 3.8 × 10^6^ time steps were needed to complete the simulation.

During the course of the simulation, Voronoi volumes were determined as described in section ‘Method details’. Exact volume determinations and gradient updates were made when contacts were formed or broken and when the magnitude of any component of Δ**R** exceeded a certain predefined threshold, viz.

(30) $$\max_{\mathrm{ij}}\left|\frac{\Delta R_{\mathrm{ij}}}{r_{0}}\right|\geq \epsilon ,$$

where *ϵ* was selected as 0.1 and 1% (as before, *r*_0_ denotes the particle radius). Whenever updates were promted by changes in Δ**R**, the exact and approximate Voronoi volumes were stored. Based on these, the relative error of the volume change was calculated as

(31) $${ɛ}_{\Delta V}=\frac{\Delta V-\Delta V_{\mathrm{approx}}}{\Delta V},$$

where Δ*V* = *V* − *V*_0_ is the exact volume change and Δ*V*_approx_ = *V*_approx_ − *V*_0_ is the approximate volume change since the last update that yielded the volume *V*_0_. Data were binned based on the magnitude of Δ*V* and the average and maximal values of |*ɛ*_Δ*V*_| were determined for each bin.

## Numerical results and discussion

**Fig. 3:**
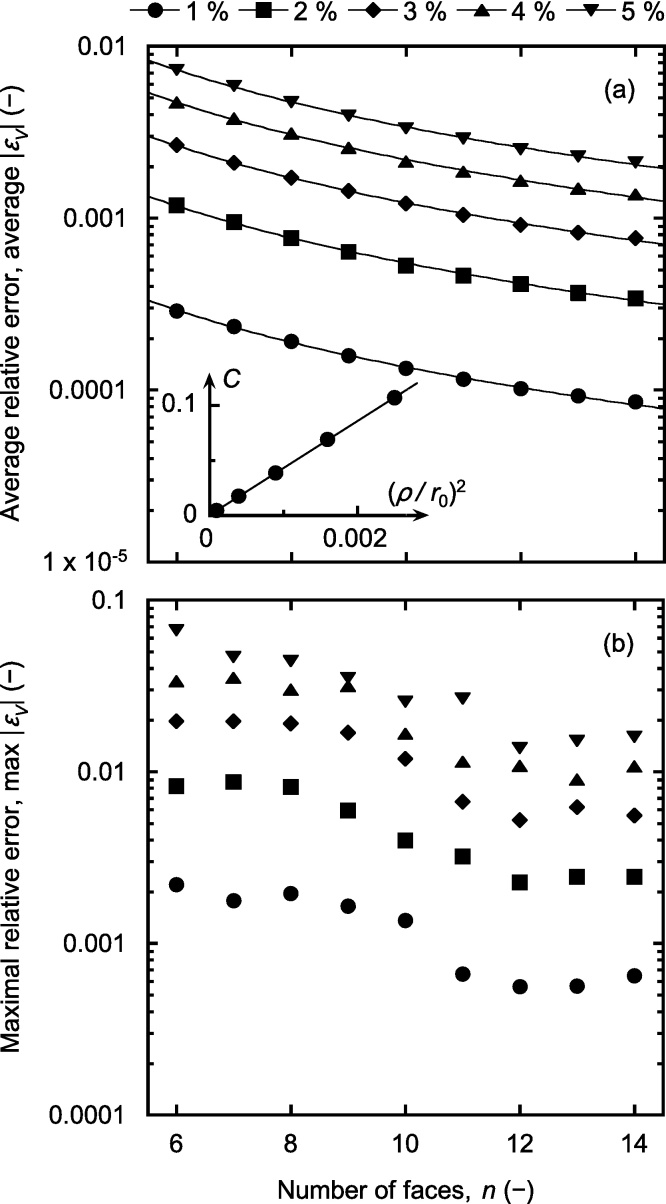
Average (a) and maximal relative errors (b) of the Voronoi volumes for the indicated values of *ρ*/*r*_0_. The solid lines in (a) represent fits of the function *C*/*n*^3/2^ to the numerical data, and the inset displays the proportionality constant *C* vs. (*ρ*/*r*_0_)^2^.

**Fig. 4:**
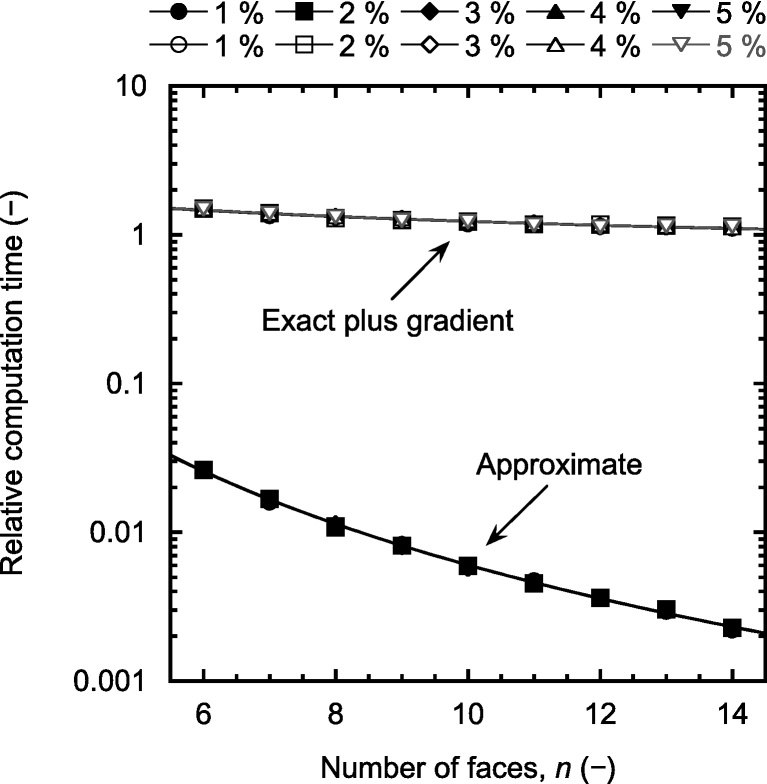
Computation times, relative to those for exact calculations, for approximate and full (i.e., exact plus gradient) calculations for the indicated values of *ρ*/*r*_0_ (the solid lines represent power-law fits to the numerical data).

The results obtained from the random polyhedra are summarised in Fig. 3, Fig. 4. The average and maximal relative errors of the Voronoi volumes, determined for five different values of *ρ*/*r*_0_ between 1 and 5%, are displayed as a function of the number of faces, *n*, in Fig. 3a and b. In general terms, both the average and maximal errors decreased with increasing *n* and the maximal error is seen to be about one order of magnitude larger than the average error. It was empirically found that the average error could be described by a function of the form *C*/*n*^3/2^, where the proportionality constant *C* depended on *ρ*/*r*_0_ (solid lines in Fig. 3a). Since the approximate Eq. (3) is first-order accurate, one expects the truncation error to be proportional to (*ρ*/*r*_0_)^2^, and the constant *C* was indeed found to be proportional to (*ρ*/*r*_0_)^2^ (inset in Fig. 3a). It is clear that an accurate approximation was obtained as long as *ρ*/*r*_0_ remained small. Specifically, a maximal error of about 0.2% was observed when *ρ*/*r*_0_ did not exceed 1% and a maximal error of about 1% was obtained when *ρ*/*r*_0_ was at most 2%.

Computation times for approximate and full (i.e., exact plus gradient) calculations are displayed as a function of *n* in Fig. 4. To enable a clearer view, all values have been normalised by the time required for the corresponding exact volume calculation (depending on *n*, this value ranged from a few seconds to about 1 min on the particular hardware used). As seen, consistent computation times were observed for all five values of *ρ*/*r*_0_. It is evident that the proposed approximation considerably reduced computation times, especially for large values of *n*, where a speedup around 450 was observed. The computational overhead introduced by gradient calculation ranged from about 50% for *n* = 6 to about 10% for *n* = 14.

**Fig. 5:**
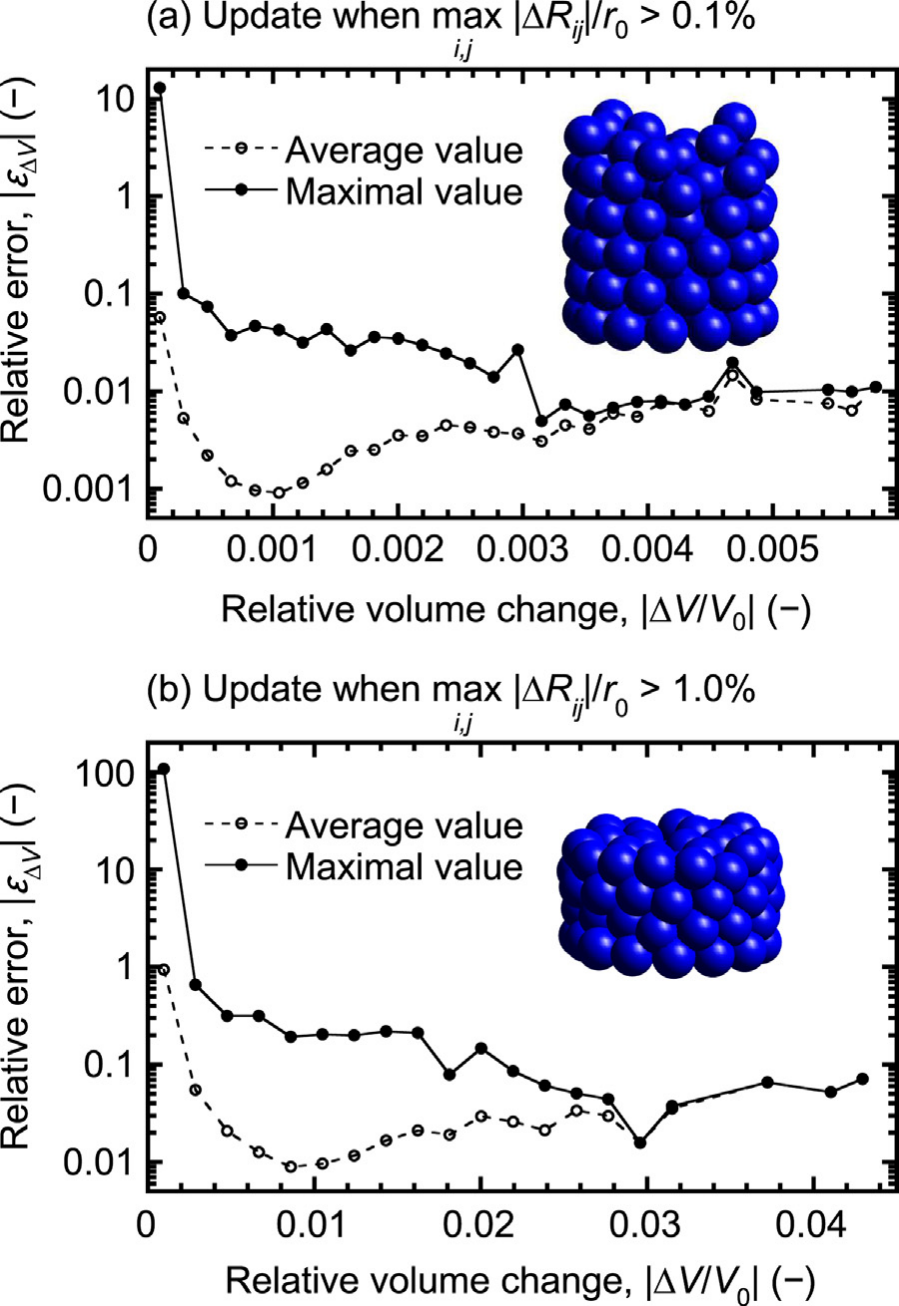
Average and maximal relative errors of the volume changes as a function of the relative volume change for (a) *ϵ* = 0.1% and (b) *ϵ* = 1%. The insets in (a) and (b) show the initial and final particle arrangements.

The results from the small-scale DEM simulations are summarised in Fig. 5, which displays the average and maximal relative errors of the volume changes as a function of the magnitude of the volume change, expressed as |Δ*V*/*V*_0_|. The insets in Fig. 5a and b show the initial and final particle arrangements. As can be clearly seen, the magnitude of the relative errors of the volume changes (Fig. 5) are considerably larger than the magnitude of the relative errors of the Voronoi volumes (Fig. 3). Moreover, the relative errors increase when |Δ*V*/*V*_0_| decreases and may, for sufficiently small values of |Δ*V*/*V*_0_|, exceed unity. This behaviour is not unexpected but is rather a consequence of the nature of the approximation. Using matrix notation and expanding the volume to second order, one obtains

(32) $$V\approx V_{0}+{\bar{\text{G}}}_{0}^{T}\Delta \bar{\text{R}}+\frac{1}{2}{\left(\Delta \bar{\text{R}}\right)}^{T}{\bar{\text{H}}}_{0}\Delta \bar{\text{R}},$$

where $\Delta \bar{\text{R}}$ and ${\bar{\text{G}}}_{0}$ represent ‘flattened’ versions of Δ**R** and **G**_0_, i.e., vectors with 3*n* components rather than *n* × 3 matrices, ${\bar{\text{H}}}_{0}$ is the corresponding Hessian matrix and the superscript ‘T’ indicates the matrix transpose. Hence, the relative error of the volume change can be approximated as

(33) $${ɛ}_{\Delta V}\approx \frac{-(1/2){\left(\Delta \bar{\text{R}}\right)}^{T}{\bar{\text{H}}}_{0}\Delta \bar{\text{R}}}{{\bar{\text{G}}}_{0}^{T}\Delta \bar{\text{R}}+(1/2){\left(\Delta \bar{\text{R}}\right)}^{T}{\bar{\text{H}}}_{0}\Delta \bar{\text{R}}}.$$

The truncation error will be of the first order but will nevertheless be small provided that $\left|{\bar{\text{G}}}_{0}^{T}\Delta \bar{\text{R}}|\gg (1/2)|{\left(\Delta \bar{\text{R}}\right)}^{T}{\bar{\text{H}}}_{0}\Delta \bar{\text{R}}\right|$. As demonstrated by the numerical simulations, this condition is generally fulfilled provided that the magnitude of all components of $\Delta \bar{\text{R}}$ are sufficiently small. Exceptions occur when the positive and negative contributions to ${\bar{\text{G}}}_{0}^{T}\Delta \bar{\text{R}}$ balance each other, in which case Δ*V*/*V*_0_ becomes small. According to our experience, the proposed method nonetheless appears to work well in practice with *ϵ* ∼ 0.1%, likely because relative changes of the order of 10^−4^ in the Voronoi volume are insignificant.

When the described method is used in DEM simulations, the overall savings in computation times will depend on how frequently the gradient needs to be updated on average. However, given the relatively modest overhead introduced by gradient calculation, significant speedups will result also for relatively frequent updates. This is substantiated by Table 1, which shows the total number of gradient updates and computational times for the small-scale DEM simulations. It might be possible to optimise the procedure used for the exact volume determination even further [7]. However, judging from the data presented in Table 1, such an optimisation would have a modest impact on the overall computational times. Moreover, the proposed local procedure circumvents the costly determination of Voronoi diagrams (or the dual Delaunay tetrahedralization) inherent in alternative methods [8].

## Additional information: background

The discrete element method (DEM), developed by Cundall and Strack [1], has established itself as the *de facto* standard technique for micromechanical simulations of granular systems in diverse application areas (e.g. [9], [10]). The method is fairly efficient, at least in relative terms, owing to the generally employed simplified contact models. Assuming mechanical independence of contacts, interparticle forces are defined in terms of certain functions of the particle overlap (e.g. [11], [12]). Although the majority of applications of the DEM have been related to dynamical processes with limited particle deformation, the method is increasingly used to study the behaviour of granular materials under confined conditions, as during manufacturing of tablets or machine parts by compression/compaction (see [9] and references therein).

Compression/compaction can often be considered as a macroscopically quasistatic process [13], characterised by extensive particle deformation. As a result, the assumption of contact independence will not be valid at high relative densities (exceeding about 0.85–0.90 for monodisperse spherical particles [14], [15]). The combined finite/discrete element method (FE/DE method) [16], [17] – sometimes also referred to as the multiparticle finite element method (MPFEM) [18] or the meshed discrete element method (MDEM) [19] – has been proposed to overcome this limitation. When the FE/DE method is used, each particle is meshed by finite elements that enable a superior representation of particle deformation, but also result in a significantly higher computational cost. The FE/DE method is highly valuable in the detailed study of systems comprising a few particles, but is unpractical for large-scale simulations due to its prohibitive computational cost. Hence simplified models for the interaction between particles under confined conditions, suitable for implementation in the DEM, are needed.

The most important ingredient in such models appears to be the constraint imposed by plastic incompressibility [17], [19], [20]. Plastic deformation can only proceed as long as there is a void space that can accommodate the displaced material. This in turn necessitates that a way be found to estimate the volume of the available void space. The most natural and promising approach seems to be to use volume estimates based on Voronoi constructions, as originally proposed by Arzt [14] and subsequently used in the DEM by Donzé et al. [19]. Since the methodology is well developed and open-source software exists, such as CGAL [21] and Voro++ [22], the main challenge is to retain the computational efficiency of the DEM despite the overhead introduced by Voronoi volume determination. Voronoi volumes can readily be determined from half-space representations using the mentioned codes, but other representations are used internally.

## Figures and Tables

**Table 1:** Number of gradient updates and computational times for the small-scale DEM simulations. For comparison, the total number of iterations was 3,773,748 and the computational time was 8192 s when exact volume determination was used.

Update limit *ϵ*	No. of updates	Computational time (s)
0.1%	148,238	2676
1.0%	45,271	2671
